# The Benefits of Supplementary Fat in Feed Rations for Ruminants with Particular Focus on Reducing Levels of Methane Production

**DOI:** 10.5402/2011/613172

**Published:** 2011-08-29

**Authors:** J. Rasmussen, A. Harrison

**Affiliations:** IBHV, Faculty of Life Sciences, Copenhagen University, Grønnegaardsvej 7, 1870 Frederiksberg C, Denmark

## Abstract

Methane (CH_4_), a highly potent greenhouse gas, has repeatedly been identified as a significant contributor to global warming. In this connection, ruminants, animals that produce large quantities of methane, have been singled out as an area for reduction with regard to their emissions to the atmosphere. In an analysis of recently published data, we identify the underlying mechanisms of methane production in ruminants and focus on the efficacy of different fat sources in terms of their ability to reduce methane production. Specific attention has been placed on *in vivo* studies involving cattle and sheep, as well as studies based on a large number of animals (>10), recorded over a longer period (>21 days), and employing reliable techniques for the quantification of methane production. Data clearly indicate that supplementary fat, given to ruminants inhibits methane production, with medium-chain fatty acids (laurin, myristic acid) as well as poly-unsaturated fatty acids (linoleic and especially linolenic acid) having a significant effect. It is also apparent that conflicting findings between individual published trials can largely be resolved when one takes into consideration differences in experimental design, the composition of the basic feeds, the fat sources used, and the number of animals involved.

## 1. Implications

The addition of supplementary fat to the diet of ruminants has been reported to effectively reduce methane production. Primarily it is the medium chain (laurin and myristic acids) and polyunsaturated (linoleic and especially linolenic) fatty acids that appear to be most efficacious. In terms of the future, and alternate cost-effective sources of such fatty acids, researchers and ruminant nutritionists might consider using the n-3 alpha-linolenic acid typically found at high levels in and readily extractable from AFA aphanizomenon flos-aquae, a type of blue green algae that grows worldwide.

## 2. Introduction

**Figure 1:**
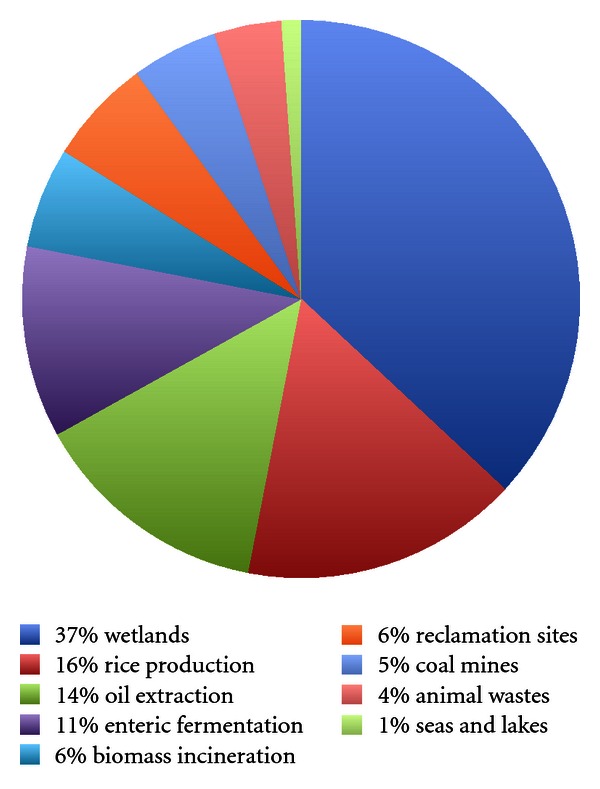
The global distribution of methane production (689 mill. tons) expressed in percent; 37% Wetlands, 16% Rice production, 14% Oil extraction, 11% Enteric fermentation, 6% Biomass incineration, 6% Reclamation sites, 5% Coal mines, 4% Animal wastes, 1% Seas and lakes. Source: [1].

There has been considerable interest in recent years in those factors that appear to contribute to global warming, as determined by an observed increase in atmospheric temperature. Moreover, global warming has thus far been linked to an increased concentration of greenhouse gases in the atmosphere, among them carbon dioxide (CO_2_), methane (CH_4_), and nitrous oxide (N_2_O) [1]. Methane is a very important greenhouse gas since it has been reported to have an effect that is 21-times greater than that of carbon dioxide in terms of global warming [2]. Furthermore, a rise in emission of methane is positively correlated with an increase in population size, to which end, currently about 70% of methane production arises from anthropogenic sources. Agricultural emissions of methane were estimated to be 10.2 million tons a year in 1990 for the EU, and this sector represents the greatest source of methane emissions within the EU [1]. Next to wetlands, the production of methane in the digestive tract of livestock is one of the most significant sources of excretion of methane worldwide. Indeed, it can be seen in Figure 1 that the global production of methane by livestock is calculated to be some 11%. 

According to a recent report by Mikkelsen et al. [3] the production of methane from the digestive tract of livestock within Denmark amounts to 133,000 tons every year (Table 1), of which cattle are responsible for 114,000 tons per year, and dairy cows some 72 tons per year. 

Ruminants have evolved a complex digestive tract, which is reliant on microbial fermentation of the organic material they consume. A byproduct of this microbial fermentation is, however, methane which is mainly eructated *via *the esophagus and released to the atmosphere through the nose. In ruminants, methane (CH_4_) is produced principally from microbial fermentation of hydrolyzed dietary carbohydrates in the rumen and hindgut, where hydrogen (H_2_), produced during conversion of hexose into acetate or butyrate, is used by methanogenic bacteria to reduce carbon dioxide (CO_2_) into CH_4_ to form energy [4]. Methane production is therefore dependent on the volatile fatty acids (VFA; acetate, propionate, butyrate) produced mainly from carbohydrate fermentation in the rumen. Feeding on high levels of neutral detergent fiber (NDF) yields a higher acetate : propionate ratio and thereby a higher CH_4_ production [5] while adding grain, rich in readily fermentable starch to a forage diet, will favour propionate production, and as a consequence, the level of CH_4_ produced will be much lower [1, 6]. 

A variety of nutritional approaches, in the form of a changed feeding strategy or use of methane inhibitors, have been investigated over the years, with a view to reducing methane emissions and to optimizing the energy metabolism of ruminants. Many methane inhibitors have been shown to be effective in reducing methane production, but most seem at present to be too expensive or have other undesirable characteristics. Supplementation of feeds with fat, especially polyunsaturated fatty acids (PUFA) and medium-chain fatty acids (MCFA), in cattle feed has been shown to significantly reduce methane emissions in cattle (for review, see the meta-analysis by [7]). These fatty acids have a toxic effect on fiber digesting bacteria, protozoa, and methanogens, and for this reason, supplementation with a fat source rich in PUFA or MCFA, to a roughage-based diet, reduces the digestibility of cell wall carbohydrates, the production of hydrogen and finally methane levels [8]. PUFA also has an inhibitory effect on methane production through direct use of hydrogen by saturation in the rumen [1, 5, 9, 10].

Thus, this review has sought to investigate and collate the known effects of different fat sources used as supplements in the feed given to ruminants, and in so doing highlight those that serve most efficiently as methane inhibitors in ruminants.

## 3. Material and Methods

In 2008, Eugène and colleagues performed a meta-analysis on the effects of lipid supplementation on methane production in lactating dairy cows. Their findings were that supplementary lipids reduced the methane production of ruminants primarily through a reduction in dry matter intake. In light of further research in this field over the last two to three years (circa 9 papers), we have chosen to reexamine the effect of supplementary fat in feed rations for ruminants and investigate its mode of action. 

This review is based on a literature study, which was undertaken to document the effect of different fat sources on the methane production of ruminants. Several search engines and reference sources were used including Agricola, Agris, CAB Abstracts, Embase, MEDLINE, Web of Science, and Google Scholar. The search criteria adopted for this selection, which are given in Table 2, were in brief, such search words as “cattle,” “methane,” “fatty acid,” “ruminant,” and “dairy cow” were used to identify possible manuscripts of relevance. In brief, attention has been given primarily to studies that reported research findings from beef cattle, dairy cows, and sheep, there after the number of animals per experiment was prioritized, the duration of the study was also taken into account, and finally selection was made based on the technique adopted for quantification of methane production. Thus, out of 14 identified papers, 7 *in vivo* experiments were short-listed, and these are outlined in Table 2. Data presented in the remaining papers, which consist of both *in vivo* and *in vitro* experiments, were found to be relevant as discussion material.

The effect of coconut oil (CO) on methane production in the rumen of beef cattle was studied by Jordan et al. [11, 12]. In experiment 1 [12] forty-one Charolais and Limosin cross-bred beef heifers were assigned to one of the following dietary treatments: 0 g, CO/d, 250 g CO/d or 250 g CM/d (copra meal containing CO), equivalent to approximately 10% of DM. The experiment lasted for 93 days and the methane production was measured twice during the trial period over a time interval of five days (days 14–18 and days 70–74). In experiment 2 [11] the effect of increasing levels of coconut oil (0; 125; 250; 375 g/d corresponding, resp., to 14, 28 and 42% of DM) on methane production and digestibility in the rumen was studied. Sixteen Charolais and Limosin cross-bred beef heifers were investigated using a 3 × 35 day trial period. Methane production was measured over a time interval of five days (days 31–35). In both studies the heifers were given a 50 : 50 forage : concentrate ratio diet *ad libitum*, comprising grass silage and barley/soya bean meal. Methane emission was measured using a modification of the SF_6_ (sulphur hexafluoride) tracer gas technique described by Johnson et al. [13]. The average daily gain was calculated, and any response in terms of digestibility was studied. An analysis of the rumen environment (microflora) was not, however, undertaken in these experiments. 

Jordan et al. [14] also studied the effect of soya oil (SO) and soya bean (SB) on methane production of young bulls. Thirty-six Charolais and Limosin cross-bred beef bulls were given a 10 : 90 forage : concentrate ratio diet, supplemented with either 0% soya, 10% soya oil or 12% whole soya bean. The trial period lasted for 103 days, and the methane production was measured 2 × 5 days (days 37–41 and days 79–83) using the SF_6_ tracer technique. The average daily gain was calculated and an analysis of the feed content as well as the rumen environment was made. 

The effect of soya oil (SO) on methane production in lambs has also been studied by Mao et al. [15]. Thirty-two Huzhou lambs were given a 60 : 40 forage : concentrate ratio diet supplemented with SO (3% of DM). The experiment lasted for 60 days and methane production was measured for three days using open-circuit respiratory chambers. Subsequently, the lambs were slaughtered and rumen samples were taken for analysis of their microflora. 

Beauchemin et al. [16] undertook an experiment with sixteen lactating cows, all of which were given a 45 : 55 forage : concentrate ratio diet, supplemented with one of three oilseed treatments: sunflower seeds (SFS), linseed oil (LO) or rapeseed (RS), to study any response in terms of methane production as well as milk yield. The experiment lasted for 4 × 28 days so that the experiment consisted of one control period with no treatment followed by 3 × 28 days in which the basal diet was supplemented with the aforementioned oilseeds (3.3% of DM). The cows were placed in two groups containing eight cows in each group. Eight ruminally cannulated cows were assigned to group 1, so that rumen samples could be taken for analysis of the microflora composition. Methane production was measured over the last week of each trial period using respiration chambers.

In an experiment undertaken by MacHmüller et al. [17] twelve Swiss White Hill lambs were assigned to six different treatments: sunflower oil (SFO), linseed oil (LO), rapeseed (RS), coconut oil (CO) and crystalline fat (not included in the data), as well as a control group which was not given added fat. The diet consisted of maize silage, grass hay and concentrate, which was supplemented with the respective lipid source (on average 56 g/kg DM corresponding to 6% of DM). The duration of the experiment was 3 × 21 days, where each trial period represented a different growth stage (resp., 30, 35, and 40 kg). Methane emission was measured over the last two days of each period using respiration cambers. An analysis of the rumen environment was carried out for all lambs and for each period (days 1, 14, and 21).

The effect of cottonseed (CS) on methane production in the rumen of lactating cows was investigated over a twelve week feeding period by Grainger et al. [18]. Fifty cows were given either a control diet or a cottonseed diet, in addition to which, each group was assigned a forage ration consisting of alfalfa hay (4.2 kg/DM/cow) and ryegrass silage (6.6 kg/DM/cow). The control group was fed 6.0 kg concentrate/DM/cow and 8% cottonseed while the treatment group was given 5.4 kg concentrate/DM/cow and 48% cottonseed. Measurements of methane production using the SF_6_ tracer technique were made on twelve cows from each dietary group on three consecutive days in weeks 2, 3, 6, 10, and 12. Samples of rumen fluid were collected from eight cows (four per diet) on two consecutive days in the same weeks, for analysis of VFA, NH_3_–N, methanogens and protozoa. Daily milk yield and composition were also measured. 

It follows that the forage : concentrate feed rate varies greatly between trials, a point that should be taken into account in relation to methane production in the rumen. Indeed, as mentioned in the introduction, methanogens are very susceptible to changes in the rumen environment, as would occur with a change in the forage : concentrate ratio. It must also be expected that feed-type, sources of fat and the percentage of fat in the diets, will all affect the amount of methane produced in the rumen and its subsequent inevitable emission to the atmosphere.

## 4. Results

Data pertaining to the different experiments outlined in the materials and methods are given in Table 2.

### 4.1. Coconut

In a study carried out by Jordan et al. [11], the effect of increasing levels of coconut oil (0; 125; 250; 375 g/day) on methane production and digestibility in the rumen was investigated. Sixteen Charolais/Limosin heifers were assigned to a 50 : 50 forage : concentrate ration diet supplemented with coconut oil. A linear reduction in the CH_4_ output occurred as the level of coconut oil in the diet increased with the greatest reduction being noted at the 375 g/day level (39%). As the level of coconut oil increased, dry-matter digestibility (DMD) decreased; however, these differences were only statistically significant at the 375 g/day level. It is therefore concluded that inclusion of coconut oil as part of a 50 : 50 silage and concentrate ration reduces methane production with no adverse effects on the DMD up to the 250 g/day level. Similar results were reported in another study of Jordan et al. [12], where forty-one beef cattle of the same cross-breed were assigned 250 g/day coconut oil as part of a 50 : 50 forage : concentrate ratio. In that case, methane production was reduced by 18% with no significant effect on digestibility. The population of protozoa and the VFA concentration declined with additional supplementation of coconut oil to the diet. 

MacHmüller et al. [17] also report a reduction in methane production in ruminants upon addition of coconut oil to the diet. Twelve lambs were assigned maize silage, hay and concentrate supplemented with coconut oil (56 g/day). The experiment lasted for 21 days and measurement of methane production was derived using respiration cambers. A 26% reduction in methane production was observed without any effect on digestibility. However, the total VFA concentration was reduced due to a decline in the acetate and butyrate concentrations. Since the propionate concentration in the rumen fluid was not significantly influenced by the different treatments, the ratio of acetate-to-propionate was, as a consequence, affected (reduced). This study also reported that lipid supplementation to the diet reduced the population of protozoa in the rumen. Finally, the different treatments had no significant effect on daily liveweight gain.

### 4.2. Sunflower

It has been shown that sunflower seed added in the feed ration given to ruminants has an inhibitory effect on methane production in the rumen [16, 17]. Beauchemin et al. [16] detected a methane reduction of 10% in a study comprising sixteen lactating cows given sunflower seed (3.3% of DM) in the ration for 28 days. Addition of sunflower seed in the ration had no effect on rumen pH or the total concentration of VFAs whereas NH_3_–N concentration was found to increase. Treatment also had no effect on milk production or milk components. According to MacHmüller et al. [17] methane production was reduced by 27% when lambs were assigned sunflower seed (6.0% of DM) in the ration. Total VFA concentration was reduced in the direction of a decline in the butyrate and acetate ratio whereas the proportion of propionate remained unaltered. However, daily liveweight gain was not significantly affected when the feed was supplemented with sunflower seed (−13 g/day on average). In both studies, an analysis of the rumen fluid showed a significant decline in the number of rumen protozoa and a significantly reduced digestibility.

### 4.3. Linseed Oil

In a study undertaken by Beauchemin et al. [16], a methane reduction of 18% was reported upon addition of linseed oil (3.3% of DM) to the diet of lactating dairy cows. Treatment had no effect on the rumen environment, since the concentration of VFA's, rumen pH, and the population of protozoa were found to be unaltered. On the other hand, digestibility was affected negatively, although milk production and milk components remained unaffected. Interestingly, though MacHmüller et al. [17] observed a lesser effect of linseed oil (6.6% of DM) on methane production (10% reduction) in lambs. They also noted that digestibility, the total concentration of VFA's and protozoa numbers in the rumen were also reduced with linseed addition to the diet. Moreover, the relative proportion of VFA changed in the direction of a decline in butyrate and acetate, whereas propionate remained unchanged. However, the daily liveweight gain was not significantly influenced (−24 g/day on average) by linseed oil supplementation to the diet.

### 4.4. Rapeseed

The effect of rapeseed (3.3% of DM) on methane production in sixteen lactating cows was studied by Beauchemin et al. [16]. The trial period lasted for 28 days and methane production was found to be reduced by 16%, without affecting digestibility. Adding rapeseed to the diet did, however, numerically reduce the population of protozoa in the rumen, although VFA concentration, rumen pH, and NH_3_–N remain unchanged. Treatment did not affect milk production or milk components. Similarly, MacHmüller et al. [17] showed a methane reduction of 19% in the rumen of lambs supplemented with rapeseed (6% of DM) in the ration. Here the treatment with rapeseed was found to have a negative effect on the number of protozoa in the rumen, digestibility, and total VFA concentration, mainly at the expense of a decline in the butyrate and acetate proportions. Daily liveweight gain was not significantly affected by rapeseed supplementation, but was numerically reduced in relation to the control group (−27 g/day on average)

### 4.5. Soya

The effect of different soya products as supplements to the feed ration of ruminants has been investigated in several studies. In an experiment by Jordan et al. [14] the effect of soya oil and soya beans on methane production were investigated in thirty-six Charolais/Limosin young beef cattle. The trial period lasted for 103 days and the animals were given a 10 : 90 forage : concentrate ratio diet. Supplementation with soya oil (10% of DM) had the greatest effect on methane production with a reduction of 40% whereas soya beans (12% of DM) resulted in a lesser reduction (25%). Mao et al. [15] reported a 14% reduction in methane production in lambs upon addition of soya oil (3% of DM) to a 60 : 40 forage : concentrate ratio diet. Moreover, soya oil supplementation resulted in a decline in rumen pH as a result of an increase in VFA concentration, whilst the population of methanogens and protozoa was inhibited, and microbial protein content increased. Finally, a decline in digestibility was observed, a change that is most likely due to the observed changes in the rumen environment.

### 4.6. Cottonseed

The effect of supplementing the diet with cottonseed, on methane production in the rumen of cows, was studied for twelve weeks by Grainger et al. [18]. Fifty lactating cows were given a forage : concentrate ratio of 10.8 kg DM/cow and 5.4 kg DM/cow, respectively, of which the concentrate comprised 48% cottonseed. A methane reduction of 23% was reported, and treatment had no apparent effect on the rumen environment, since no negative effect was observed in terms of the VFA concentration, protozoa number, methanogen number, or ammonia level. On the other hand supplementation with cottonseed decreased the milk yield (10%), had no effect on milk fat concentration, but did decreased concentration of milk protein (5%) and lactose (11%).

## 5. Discussion

Methane produced by microbial fermentation in the rumen is not only associated with a loss of energy in ruminants, it has important environmental consequences, and a reduction is therefore advantageous from an economic as well as an ecological standpoint. Supplementation of ruminant diets with different plant oils and with beans has been shown by several studies to reduce methane emission. 

### 5.1. Methane Inhibitors

A series of methane inhibitory compounds fed with the ration have also been shown to influence the production of methane in ruminants, either by influencing the rumen microflora [19], or by sequestering hydrogen [16, 20, 21]. These additives may take the form of halogenated methane analogues (including such compounds as chloroform, aminchloral, trichloroacetamide, trichloroethyl, and bromochloromethane) or maybe ionophores (e.g., antibiotics; [1]), or alternatively biologics (e.g., viruses, bacteriocins, yeasts; [9]) as well as organic acids such as fumarate and malate, and propionate precursors, or indeed substances acting as a sink for metabolic hydrogen. Unfortunately, though many of these chemical compounds are not acceptable for general use in the agricultural industry. Ionophores, for example, are undesirable because of problems with resistance, and halogenated methane analogues are typically directly toxic. 

Biological methods are also problematic, being mostly reliant on genetic engineering and very often being technologically crude. Indeed, the use of biological resources also requires a change in attitude towards the acceptance of the use of genetically modified organisms, and extensive testing needs to be carried out to ensure there are no adverse effects of such treatments before genetically modified organisms can be introduced to an open and biologically active environment like the rumen. 

In a recent review, Beauchemin and colleagues [22], highlighted short-term nutritional management strategies capable of reducing methane production, which included supplementation with saponins, tannins, yeast cultures, and fibre digesting enzymes. However, more promising methods, which could also be deemed acceptable for general use in an agricultural setting, are bacteriocins such as nisin, as well as hydrogen-precursor substances such as fumarate or malate. Indeed, based on the findings of an *in vitro *study, nisin can be expected to reduce methane production by approximately 36% [23]. Likewise, according to findings by Newbold et al. [24] addition of fumarate may reduce methane production by 17%. Importantly, such hydrogen-precursor resources such as fumarate, which are natural intermediary products in rumen fermentation processes, also seem to pose relatively few problems with regard to ethical and consumer issues. If these substances can be obtained at a favorable price then this type of technology could become widespread, since such substances could be readily incorporated into commercial feed compounds.

### 5.2. Dietary Fats and Rumen Fermentation

Different types of supplementary fats added to the feed ration have also been shown to inhibit methane production, but their effectiveness depends on multiple factors. It is now apparent that the type of ruminant selected for supplementary fat trials can partly explain variations between trials, since the fermentation patterns and the population of microbes within the rumen differ between cows and sheep. This particular point was highlighted in the trials investigating the effects of supplementary coconut oil, since the population of protozoa was reduced and the proportion of acetate + butyrate decreased in lambs [17] whereas there was no effect on microbes and the proportion of VFAs in cattle [12]. Similar results have been observed in lambs given soya oil, where the result was a decrease in rumen protozoa, methanogens and digestibility and an elevated VFA production [15] whereas there was no effect on VFA production or microbe numbers in cattle given soya oil [14]. Moreover, the fermentation patterns in cattle and lambs were reported to react in opposite ways upon supplementation with sunflower seed, linseed oil, and rapeseed, respectively [16, 17, 20]. The type and level of feeding also has an influence on the fermentation patterns and microbial balance in the rumen. For example, an increased level of NDF results in an increased acetate : propionate ratio in the rumen, and as a consequence methane production is elevated [5, 25] whereas an increased level of starch in the diet changes the fermentation pattern towards higher levels of propionate and thereby serves to decrease methane production [1, 6]. Other factors that might conceivably affect the results of such trials are; (1) the duration of the experiment, (2) the number of animals recruited, and (3) the use of different methods of quantification of methane emission. Thus, as a result of the considerable variation between experimental designs, it is difficult to draw firm conclusions on the impact of fat supplementation on methane production with only a cursory review of the available literature.

### 5.3. Methane Inhibitory Effects of Different Dietary Fats

Supplementation with coconut oil in the diet reduces the production of methane in the rumen. Coconut oil is rich in MCFA, which as several studies have shown, are effective methane inhibitors [26–28], indeed the MCFA content of coconut oil most likely explains its inhibitory effect on methane production in ruminants. Experimentally, increasing the level of dietary coconut oil results in a linear reduction in methane production in beef cattle, where supplementation of 250 g/day (10–28% of DM) and 375 g/day (42% of DM) induced in a reduction of 18–21% and 39%, respectively [11, 12]. Similarly, supplementation with coconut oil (7% of DM) in the diet of lambs causes a reduction in methane production of some 38% [29]. Consequently high levels of coconut oil (375 g/day) in the diet decrease the digestibility and DMI whereas supplementation with lower levels (250 g/day) has no influence on digestibility or DMI. In support of which, MacHmüller et al. [17] reported a methane reduction of 26% in lambs without affecting digestibility. In the later, treatment decreased the population of protozoa as well as the proportion of acetate and butyrate whereas rumen microbes and the proportion of VFA's in cattle remained unchanged [12]. 

The effect of sunflower bean, linseed oil, and rapeseed on methane production in cows and lambs has been studied by Beauchemin et al. [16, 20] and MacHmüller et al. [17], respectively. Supplementation of sunflower bean to the diet decreased methane production in lactating cows and lambs by 10% and 27%, respectively, whilst supplementation with linseed oil resulted in a reduction of 18% in cows and 10% in lambs. Consequently, in both cases dietary supplementation had a negative effect on digestibility, reflecting a direct inhibition of the cellulolytic bacteria. A decline in daily methane production in cows can be further attributed to a decrease in DMI. Supplementation with rapeseed reduced methane production by 16% in cows without affecting digestibility whereas a methane reduction of 19% in lambs resulted in a decreased digestibility. The population of protozoa was uniquely decreased upon supplementation with sunflower bean and rapeseed, whereas this occurred only with cows given linseed oil. Rapeseed has a high content of MUFA (C18 : 1) whereas sunflower bean and linseed oil are both rich in PUFA, yet differ in that sunflower bean is rich in C18 : 2 whilst linseed oil is rich in C18 : 3, which may explain in part some of the variation in terms of their effects in reducing methane production in ruminants. In support of which, Dohme et al. [26] showed a methane reduction *in vitro* upon incubation of C18 : 2 with rumen fluid of some 25%, which is consistent with a reduction in methane production of 27% *in vitro* in lambs given sunflower bean [17]. Finally, Martin et al. [30] observed a reduction in methane levels of 12–64% upon addition of different types of linseed oil in cows. The linseed oil types differed in their form of processing, which indicates that heat treatment, pelleting, and other processing steps may further complicate/influence the efficacy of these natural lipid methane inhibitors. 

Likewise, results differ in terms of the form of supplemented soya products (rich in C18 : 2) to the diet of ruminants. Jordan et al. [14] showed a methane reduction in beef cattle (bulls) of 40% and 25% upon supplementation with soya oil and soya bean, respectively. Infact, this huge difference may be due solely to the higher fatty acid content of soya oil compared to that of soya beans. Mao et al. [15] observed a somewhat smaller reduction (14%) in methane levels in lambs given soya oil. 

Supplementation with cottonseed in ruminants over a twelve week period, reduced methane production by 23% in lactating cows [18]. Cottonseed has a relatively high C18 : 2 content too, perhaps explaining why the effect of cottonseed is comparable to that of sunflower bean (27%) and that of soya bean (25%). 

Finally, it appears from the diverse research data that has been collated in this paper, that digestibility is reduced upon the addition of some fatty acids to the rumen while there are no effects with other types of fatty acids. This observation suggests that the microbial ecosystem may be influenced by some fatty acids while others may only have an inhibitory effect on say methanogens [17]. Alternatively, some supplementary fats may reduce methane production indirectly through a reduction in the intake of OM by effecting a reduction in DMI, digestibility, or both [30].

## 6. Conclusion

All things taken into consideration, addition of supplementary fat to the diet of ruminants can effectively reduce methane production. The effect of supplementary fats appears to be two-fold, the first acting through inhibition of the activity/viability of the cellulolytic bacteria in the rumen, and the second, as a consumer/binder of hydrogen. As a consequence, supplementary fats typically result in a reduction in the digestibility of cell wall carbohydrates, leading to decreased production of acetate and an increased propionate to acetate + butyrate ratio, which in turn decreases the production of hydrogen and thereby methane.

## Figures and Tables

**Table 1:** Yearly production of methane from livestock in Denmark.

Source	Livestock category	1000 tons methane/year
Digestive system	Dairy cows	72
	Cattle	114
	Small ruminants	1,4
	Horses	3,7
	Swine	14
	Total livestock	133

Livestock waste	Cattle	12
	Swine	33
	Other livestock	2
	Total livestock	47

In total, livestock produce 133 × 1000 tons of methane (CH_4_) every year. Cattle represent 86% of this production, and small ruminants represent just 1%. Source: [3].

**Table 2:** Collated data pertaining to the effect of diverse dietary fatty acids on methane production in large and small ruminants.

Art.nr.	Animal	*n*	Duration	Ration	Fat source (% of DM)	Results												Method	Conclusion	References
				Forage/conc.		Methane (%)	NH_3_	VFA	Propionate	Acetate	Butyrate	Micro protein	Protozoa	Methanogens	pH	DMI	Digestibility			
1	Heifers	41	93 d	50/50	CO (10%)	−18		↓	n.e.	n.e.	n.e.		n.e.			n.e.	n.e.	SF_6_	CO: 28% reduction	[12]
	Charolais/				250 g/d	−14.9													digestibility n.e.	
	Limosin				CM (10%)			↓	n.e.	n.e.	n.e.		n.e.			n.e.	↓			
	*in vivo*				250 g/d															
2	Heifers	16	35 d	50/50	CO (14%)	−13.5										n.e.	n.e.	SF_6_	Linear reduction with	[11]
	Charolais/				125 g/d														increasing amount	
	Limosin				CO (28%)	−20.5										n.e.	n.e.			
	*in vivo*				250 g/d															
					CO (42%)	−39										↓	↓			
					375 g/d															
3	Bulls	36	103 d	10/90	SO (10%)	−40		n.e.	n.e.	n.e.	n.e.		n.e.			n.e.		SF_6_	SO: 40% reduction	[14]
	Charolais/				SB (12%)	−25.3		n.e.	n.e.	n.e.	n.e.		n.e.		↓	↓			digestibility n.e.	
	Limosin																			
	*in vivo*																			
4	Lac. cows	50	12 wk	70/30	CS (48%)	−23	n.e.	n.e.					n.e.	n.e.		n.e.		SF_6_	Methane ↓ (−23%)	[18]
	*in vivo*																		digestibility n.e.	
5	Lac. cows	16	4 × 28 d	45/55	SFS (3.3%)	−10	↑	n.e.	n.e.	n.e.	↓		↓		n.e.	↓	↓	RC	LO : 18 % reduction	[20]
	*in vivo*			TMR	LO (3.3%)	−18	n.e.	n.e.	n.e.	n.e.	↓		n.e.		n.e.	↓	↓		digestibility ↓	
					RS (3.3%)	−16	n.e.	n.e.	n.e.	n.e.	↓		↓		n.e.	n.e.	n.e.		RS : 16% reduction	
																			digestibility n.e.	
6	Sheep	32	60 d	60/40	SO (3%)	−14	↓	↑	n.e.	n.e.	n.e.	↑	↓	↓	↓*		↓	RC	Methane ↓ (−14%)	[15]
	Huzhou																		digestibility n.e.	
	*in vivo*																			
7	Sheep	12	3 × 21 d	60/40	CO (6%)	−26		↓	n.e.	↓	↓		↓				n.e.	RC	SFS : 27% reduction	[17]
	S.W.H.				RS (6%)	−19		↓	n.e.	↓	↓		↓				↓		digestibility ↓	
	*in vivo*				SFS (6%)	−27		↓	n.e.	↓	↓		↓				↓↓		CO : 26% reduction	
					LO (6%)	−10		↓	n.e.	↓	↓		↓				↓		digestibility n.e.	
8	Bulls	16	21 d	75/25	SFO (5%)	−22		n.e.	↑	↓					n.e.		↓	RC	Methane ↓ (−22%)	[31]
	Holstein				400 g/d														Digestibility ↓	
	*in vivo*																			
9	Lac. Cows	36	319 d	50/50	CS (4%)	n.e.										↑		SF_6_	n.e. probably too	[32]
	Holstein			TMR	CS (5.6%)	n.e.										↑			small a dose	
	*in vivo*				RS (4%)	n.e.										↑			lacks hydrogenation	
					RS (5.6%)	n.e.										↑			in the rumen	
10	Lac. Cows	12	18 d	60/40	C14 : 0 (5%)	−36										n.e.	↓	RC	Methane ↓ (−36%)	[27]
	Holstein			TMR															Digestibility ↓	
	*in vivo*																			
11	Lac. Cows	8	4 wk	65/35	LO (5.7%)	−64										↓	↓	SF_6_	LO : 64% reduction	[30]
	Holstein				ELO (5.7%)	−38										↓	↓		Digestibility ↓	
	*in vivo*				CLO (5.7%)	−12										n.e.	↓			
12	Sheep	8	21 d	60/40	CO (7%)	−38		n.e.	n.e.	n.e.	↓				n.e.			RC	Methane ↓ (−38%)	[29]
	Huzhou																		Digestibility n.e.	
	*in vivo*																			
13	*in vitro*		24 h	60/40	C12/C14														Greatest effect with	[28]
	1 cow				10 : 20	−50		n.e.	n.e.				n.e.	↓	n.e.				raised C12/C14 ratio	
					15 : 15	−87		n.e.	n.e.				n.e.	↓	n.e.				(−96%). Reduction in	
					20 : 10	−96		n.e.	n.e.				n.e.	↓	n.e.				methanogens	
14	Rusitec	4	10 d	60/40	C8 : 0 (5%)	n.e.	n.e.	n.e.					↓	n.e.	n.e.		↓		MCFA : 18% reduction	[26]
	1 cow				C10 : 0 (5%)	n.e.	n.e.	n.e.	↓				↓	n.e.	n.e.		↓		PUFA : 25% reduction	
	*in vitro*				C12 : 0 (5%)	−18	n.e.	n.e.					↓	↓	↓		↓			
					C14 : 0 (5%)	−18	n.e.	n.e.					n.e.	↓	n.e.		↓			
					C16 : 0 (5%)	n.e.	n.e.	n.e.					n.e.	n.e.	n.e.		n.e.			
					C18 : 0 (5%)	n.e.	n.e.	n.e.					n.e.	n.e.	n.e.		n.e.			
					C18 : 2 (5%)	−25	n.e.	n.e.					↓	↓	↓		n.e.			

CLO = crude linseed; CM = copra meal based concentrate with 250 g of CO/d from copra meal; CO = coconut oil; CS = cottonseed; DMI = dry matter intake; ELO = extrudedlinseed; LO = linseed oil; MCFA = medium chain fatty acid; n.e. = no effect; NH3 = Ammonia; PUFA = polyunsaturated fatty acid; RC = respiration chamber; RS = rapeseed;SB = soybean; SFS = sunflowerseed; SF_6_ = sulphur hexafluoride tracer technique; SFO = sunflower oil; SO = soya oil; S.W.H. = Swiss White Hill; TMR = total mixed ration;VFA = short chain fatty acids.
